# Measuring individual hierarchy of anxiety invoking sports related activities: development and validation of the Photographic Series of Sports Activities for Anterior Cruciate Ligament Reconstruction (PHOSA-ACLR)

**DOI:** 10.1186/s12891-017-1643-9

**Published:** 2017-07-04

**Authors:** Wim van Lankveld, Nicky van Melick, Bas Habets, Eefje Roelofsen, J. Bart Staal, Robert van Cingel

**Affiliations:** 1 HAN University of Applied Sciences, Research group Musculoskeletal Rehabilitation Nijmegen, Kapittelweg 33, Nijmegen, The Netherlands; 2 Radboud university medical center, Radboud Institute for Health Sciences, IQ healthcare, Nijmegen, The Netherlands; 3 Sport Medical Centre Papendal, Arnhem, The Netherlands

**Keywords:** Anterior cruciate ligament reconstruction (ACLR), Kinesiophobia, Fear of harm/movement/injury, Validation

## Abstract

**Background:**

Fear of harm (FoH) after Anterior Cruciate Ligament Reconstruction (ACLR) should be addressed in physical therapy as it hampers return to sports. However, there are no instruments assessing FoH specific for ACLR. The objective of this study is to describe the development and measurement properties of the Photograph Series of Sports Activities for ACLR (PHOSA-ACLR) measuring ACL injury related FoH.

**Methods:**

Based on literature and opinion of physical therapists with extensive experience in ACLR treatment, photographs depicting FoH inducing situations in ACL injury were considered for inclusion in the instrument. For each photograph the patients is asked to report perceived harmfulness. The set of photographs was completed by two samples of patients with ACLR: 1 cross-sectional sample (*n* = 55), and 1 test-retest reliability sample (*n* = 58). Internal consistency and structural validity were assessed in 109 patients. In 58 patients criterion validity was assessed by calculating pearson correlations with the Tampa Scale of Kinesiophobia (TSK). Correlations with self-reported knee function (KOOS and Lysholm score), and Knee Self-efficacy Scale (K-SES) were computed for hypothesis testing. Test-retest reliability was determined in a group of 55 patients, assessed twice with 1 week between assessments.

**Results:**

Twelve photographs depicting sports related movements that are likely to invoke FoH after ACLR were selected. Two items were deleted because of lack of discrimination. The remaining 10 items were included in the PHOSA-ACLR, and the scale showed excellent internal consistency (Cronbach’s Alpha is .95). Items reflected one dimension, and was strongly correlated with TSK (*r* = .59). A priori formulated hypotheses are confirmed and test-retest correlation was excellent (ICC = .86).

**Conclusion:**

The PHOSA-ACLR showed acceptable measurement properties. The PHOSA-ACLR gives specific information about fear invoking sports situations that are not measured by other kinesophobia measures. Therefore, the PHOSA-ACLR might be a valuable additional tool in rehabilitation of ACLR patients. Additional research is needed to determine responsiveness to change.

**Electronic supplementary material:**

The online version of this article (doi:10.1186/s12891-017-1643-9) contains supplementary material, which is available to authorized users.

## Background

Anterior Cruciate Ligament (ACL) rupture is a sports injury occurring most often in sports that include pivoting movements of the knee, such as soccer, basketball, football, handball, and skiing. To return to previous sports levels, ACL reconstruction (ACLR) is a frequently performed intervention in these athletes. It is estimated that the number of ACLR for the USA is 120,000–200,000 a year [1]*,* and 8000–9000 in the Netherlands [2]*.* Recovery after ACLR to activity of daily living (ADL) is observed after 9 to 16 weeks of rehabilitation [3]*.* One year after the injury, only half of the athletes undergoing ACLR have returned to sports at their pre-injury level [4–6]*.*

Psychological factors have been identified to influence return to sports in athletes after ACLR [7]*.* Cognitive factors influencing return to sport include internal Health Locus of Control [8] and Self-efficacy [9]*.* In the affective dimension, fear of harm (FoH) through movement has been extensively researched. Fear of movement or re-injury (kinesiophobia) during rehabilitation has negative consequences for the rehabilitation process [10]*.* It has been suggested that behaviour associated with fear of movement hampers return to sport [11]*.* Patients returning to previous levels of sports report lower levels of fear of movement [12–14]*,* and half of the people who did not return to pre-injury or competitive sports levels were afraid of injury [5, 13, 15]. Fear of injury or lack of confidence in the knee are most frequently cited as the reasons for not returning to sport [16]*,* and fear-of-movement is recognised as a crucial element in the rehabilitation after ACLR [7]. Addressing psychological factors including fear of injury and confidence in ACLR rehabilitation can help people return to their preinjury sport or recreational activity [16]. Taking the above into consideration, a biopsychosocial approach is needed to address the interrelated cognitive, affective, and behavioural factors that impact return to sport after injuries [17]*.*

Fear for specific movements can be addressed using methods of graded exposure [18]. Graded exposure guides the patient through an individualized hierarchical series of fear eliciting movements and activities that they have been avoiding. Starting with the least fear inducing activity, an opportunity is created for the patient to correct catastrophic misinterpretations of wrongful association between movements and harm. Therefore, it might be expected that graded exposure in ACLR rehabilitation can help overcome fear of new injury and not trusting the knee, which are recognised as two of the main reasons for not returning to pre-injury levels of sports [4]. For such an individual approach a diagnostic tool is needed to determine a hierarchy of perceived harmfulness of different physical activities and movements specific for ACLR. FoH in ACLR research is most frequently assessed using the Tampa Scale of Kinesiophobia (TSK) [19]*.* A less frequently used FoH measure specific for ACLR is the ACL-Return to Sports Injury Scale (ACL-RSI) [20]. However, both scales do not provide information about specific movements or activities that induce fear of re-injury in the individual. As a consequence, constructing an individual hierarchy in fear eliciting activities is impossible. To be able to determine such individual hierarchy of fear eliciting activities after ACLR a new tool is needed. In low back pain, an instrument to determine the hierarchy of fear for movements is the Photograph Series of Daily Activities (PHODA). The PHODA measures the patient’s judgement about the harmful consequences of certain movements of activities of daily life depicted by photographs [21, 22]*.* However, the PHODA is specific for patients with chronic low back pain, and does not include ACL specific fear inducing movements. Therefore, there is a need for a diagnostic tool to determine perceived harmfulness of specific sports related activities that induce fear of re-injury in patients with ACLR. Such an instrument specifically for ACLR can be used as a guiding instrument for treatment decisions, and as a measurement instrument to evaluate the effects of treatment in this patient group.

For that purpose, we developed the Photographic Sports Activity-Anterior Cruciate Ligament Reconstruction (PHOSA-ACLR), which consists of 12 photographs depicting sports activities assessing fear of movement, and allows to establish an individual hierarchy of sports related fear inducing activities. This study reports on the development of the PHOSA-ACLR, and it’s measurement properties.

## Methods

Development of the PHOSA-ACLR was guided by the COnsensus-based Standards for the selection of health Measurement Instruments (COSMIN) [23]. The following COSMIN measurement properties were evaluated: content validity, structural validity, internal consistency, construct and criterion validity, reliability, measurement error, and hypothesis testing.

### Step 1: Development of the PHOSA-ACLR (content validity)

*Content validity* shows that the content of the scale under construction is relevant and comprehensive for future users [23]. The aim of the PHOSA-ACLR is to assess fear of harm (FoH), for specific sports activities after ACLR. Rather than asking the patient to rate a set of statements to assess kinesophobia, the instrument uses photographic images depicting sports activities that are likely to invoke fear of harm or re-injury. The instrument was designed to reflect one single construct (i.e. “perceived harmfulness”), and each item is considered an effect indicator. Items to be considered for inclusion in the instrument should fulfil some requirements. Firstly, activities that were identified as the most important causes of ACLR should be depicted in the instrument as these items are most likely to invoke anxiety. An ACL rupture most often occurs during jumping, changing direction, running, sudden stoppage, and overextension of the lower leg [24]. Secondly, to be applicable to all patients after ACLR, the activities depicted should be easily recognisable for all athletes. Therefore, activities that were sports specific and not readily recognisable for all patients were not considered for inclusion. Finally, the new instrument should be as short as possible to enhance acceptance in physical rehabilitation practice. Based on these requirements the authors described a number of sports related activities to be considered for inclusion as items in the new instrument. A limited number of 20 sports activities were described by the authors with a background in ACLR rehabilitation (BH, RvC). These activities were discussed with two other physical therapists with at least 10 years of experience with ACL rehabilitation. From the initial 20 activities described, 12 activities were selected to be included in the PHOSA-ACLR. Eight items were excluded due to overlap. The following 12 sport related activities were depicted in photographs (in that order): running, landing after a jump, squats, lateral lunging, single leg jump, sliding, sudden deceleration (stop), hop, lunge, start to sprint, jumping and landing on a trampoline, and pivoting movement (switching direction). Students from the HAN University of Applied Sciences staged these activities in a real life environment in the gym. For each activity different photographs were made of the enacted activity, and the first author chose the image best representing that activity (for the PHOSA-ACLR see Additional file 1).

The resulting PHOSA-ACLR consists of 12 photographs depicting sports related movements that can invoke FoH after ACLR. Above each photograph, the following instruction was given: Score the activity depicted in the photograph below from 0 to 10, where 0 is “not harmful at all” and 10 is “extremely harmful”. For the first 18 ACLR patients included in the study a few additional questions were added related to the content of the PHOSA. They were asked to indicate for each item if they could relate to the depicted situation. Finally, patients were asked if they missed some activities that in their opinion should be included.

### Step 2: Measurement properties of the PHOSA-ACLR

#### Patient samples and selection

Two different samples of patients were used as part of two distinct student projects. All patients were asked to participate in the study by physical therapists familiar with ACLR treatment, who were affiliated with the HAN University of Applied Sciences. These physical therapists were informed about the study and received information in writing on the content of the study as well as general inclusion criteria: ACLR, aged between 18 and 55, and able to communicate in Dutch. Patients were informed of the study by their physical therapist, either face-to-face or by social media (twitter, face-book). Patients had to contact the researchers by e-mail to indicate their willingness to participate in the study. Participants were asked to give informed consent after being informed on the content of the study. If patients agreed to participate in the study they received an e-mail with a link to an online survey tool on “http://www.thesistools.com” (ThesisTools, Liessel, Belgium). Thesistools allows online anonymous data collection. Data assessed using ThesisTools were not shared with any third party. Data for the test-retest condition were assessed using traditional paper questionnaires.

The first sample of patients was recruited to participate in the internal consistency and validity sample if they had undergone primary ACL reconstruction more than 3 months ago, but no longer than 3 years ago. Patients with a known ACLR were invited by direct referral form two physical therapists working in Sports Medical Center (Sports Medical Center Papendal, Arnhem, The Netherlands; and Funqtio, Steyl, the Netherlands), or from primary care physical therapists in the Nijmegen area. A total of 72 participants were included. Only complete cases (*n* = 58; 81%) were included in the analysis. The second group of patients was recruited to assess test-retest reliability. Patients were recruited from the Sport Medical Centre Papendal Arnhem, The Sint Maartenskliniek Sports Medical Centre Nijmegen, and Funqtio, Steyl, the Netherlands. Only patients were included who had undergone successful reconstruction >4 weeks ago. For this sample 73 patients were invited, and 55 patients participated. When patients indicated their willingness to participate, they were phoned and given verbal information about the content of the study, as well as information in writing.

#### Measurements

In both samples age and gender were assessed as well as time since reconstruction (months). The sample used to determine Internal Consistency, criterion and structural validity completed a number of additional questionnaires.

Self-reported knee function was assessed using the Lysholm scale and the Knee injury and Osteoarthritis Outome Score (KOOS). The Lysholm scale is a valid and reliable eight-item questionnaire used to evaluate knee function after knee ligament injury [25]. The questionnaire covers 8 dimensions: pain, locking, swelling, instability, stair climbing and limping, using support, and squatting. Total score is computed by summing the scores on the 8 dimensions. The total score ranges from 0 to 100 where 0 depicts the worst possible score and 100 the best possible score. The total score can be interpreted as follows: poor <64; fair 65–83; good 84–94; excellent 95–100. The KOOS is a reliable questionnaire to assess the patient’s opinion about the knee and associated problems [26]*.* The KOOS has 42 items, which are divided in five sections: Pain, Other Disease- Specific Symptoms, ADL Function, Sport and Recreation Function, and knee-related Quality of Life. All items are scored from 0 to 4 where 0 = No problems and 4 = extreme problems. For each scale the scores were recoded from 0 to 100, with 100 depicting no problems. For this study the Dutch version was used [27]*.*

Kinesophobia was assessed using Tampa scale of Kinesiophobia (TSK) [19]. The TSK measures fear of movement or (re)injury with 17 items scored from 1 to 4 where 1 = strongly disagree and 4 = strongly agree. The Dutch version of the TSK was used for this study [28, 29]. A score on the TSK above 37 indicates fear of movement.

Self-efficacy related to knee function after ACLR was assessed using the “Knee Self-Efficacy Scale” (K-SES). The scale measures how certain the patient is about the execution of certain activities, despite pain or discomfort. The K-SES measures 4 domains of self-efficacy: daily activities; sports activities; knee functions tasks; knee function in the future [9, 30]. Permission for the cross cultural adaptation of the K-SES was obtained from the developer (personal communication, Dr. Thomee). For this cross cultural adaptation in Dutch the method described by Beaton was used [31]. This method uses forward and back translation with two independent bilingual translators for each step.

Finally, Health Locus of Control was assessed using the Multidimensional Health Locus of Control Scale (MHLC) [32]*.* This scale measures locus of control in three dimensions: internal, external, and physician locus of control. For this study the Dutch version was used [33].

#### Statistical analysis

For studies into measurement properties of questionnaires, a sample size of >100 is required, and in determining test-retest reliability a sample size of 50 is adequate [34]. All data except for the test-retest assessments were sampled using Thesis tools, and completion of data entry is only possible when all data are entered. As a consequence, there were no missing values. In the test-retest sample only 2 questionnaires included 1 missing item. These missing items were replaced with the average score of that individual on all other items. All analyses were done using SPSS version 20 (IBM Corporation). A *p* value ≤0.05 was used as an indication of statistical significance. Correlations were interpreted as small (*r* = .10), medium (*r* = .30), and large (*r* > .50) [35].

Descriptive statistics are given for each of the individual PHOSA items. Items with high response on the extremes (>50%) do not sufficiently discriminate between respondents and were excluded from further analysis. As POSA items are considered to reflect one underlying construct, *structural validity* was analysed using Principle Component analysis [36]. First, sampling adequacy was determined. The Kaiser-Meyer-Olkin (KMO) was calculated to determine if the variables included in the scale depict a common factor. A KMO value > .08 is considered good, indicating that a Principal Component analysis is warranted. Next, the Bartlett test of sphericity was conducted. When significant, the test shows that distinct items can be summarized in underlying factors. Finally, the number of underlying factors in the PHOSA-ACLR was determined using Principal Component analysis using Varimax rotation and maximum likelihood extraction. It is expected that Principal Component analysis will result in one underlying factor based on the Kaiser criterium (Scree Value >1). *Internal consistency* of the PHOSA-ACLR scale was determined using Cronbach’s alpha to assess the degree of the interrelatedness among the items. Total PHOSA-ACLR scale score was computed by averaging the item scores. The Kolgomorow-Smirnow test is used to test for univariate normal distribution of the scale score. *Criterion validity* of the PHOSA-ACLR was determined by calculating the pearson correlation with TSK, as TSK might be considered to be a gold standard for the assessment of kinesophobia.

*Reliability and Measurement error* of the test was determined in a group of patients with stable outcome that completed the PHOSA-ACLR twice with an interval of 1 week. Mean difference between test and re-test item scores were calculated with corresponding 95% confidence interval (CI). Intra Class Correlation (ICC) between both assessments was calculated with corresponding 95% CI, to determine absolute agreement between assessments. The random effects model was used. An ICC above 0.75 is considered excellent [37]*.* Standard error of measurement (SEM) was computed by dividing the SD of the mean difference between both assessments (SDdiff) by √ 2 [38, 39].

#### Hypothesis testing

To determine construct validity, a number of hypothesis have been formulated about the unidimensionality of the scale, and the relation with other variables in the study. These hypothesis are given in Table 1. The construct validity is good when >75% of the a-priori formulated hypothesis about the relation of the construct with other theoretically derived constructs are confirmed [39].

**Table 1:** Hypothesis that were tested

1	The PHOSA-ACLR items will reflect 1 underlying dimension.
2	A large positive correlation (*r* > .50) is expected between PHOSA-ACLR and TSK.
3	PHOSA-ACLR score is independent of gender and age.
4	PHOSA-ACLR score is weakly related (*r* < .50) to time since reconstruction.
5–10	PHOSA-ACLR will have moderate to large reversed correlations (*r* < −.50) with knee outcome scores (Lysholm Total and 5 KOOS dimensions).
11–13	PHOSA-ACLR will have moderate to large reversed correlations (*r* < −.50) with all four K-SES subscales.
14–16	PHOSA-ACLR will have small to medium correlations (*r* < −.30) with MHLC subscales
17	PHOSA is significantly correlated to TSK after controlling self-reported knee function

## Results

*Content validity* was addressed by asking the first 18 respondents to judge the items for their relevance. None of the items was judged to be redundant, and patients reported that they could relate to the depicted situations.

To determine *structural validity* and *internal consistency* and PHOSA items scores of the 2 patient samples were combined. The sample comprised 113 patients (average age 26.5 year, proportion males = 58%; average month since reconstruction = 9.5 (SD 9.2, range 1–48). Table 2 shows the mean scores on the 12 PHOSA-ACLR items and the standard deviation. Items are numbered as they are presented to the patient. In the table, items are rank ordered in increased average perceived harmfulness, and quartile scores are given. The PHOSA-ACLR scale score is the average of the 12 items.

**Table 2:** PHOSA-ACLR item dispersion (*N* = 113)

	Observed range	Scores at Percentile Points			Mean (SD)
		25%	50%	75%	
3. Squats.	0–10	0	1	3	1.9(2.5)
9. Lunge.	0–10	1	1	3.5	2.3(2.5)
1. Running	0–10	1	2	5	3.1(3.2)
10. Start to sprint.	0–10	1	3	5.5	3.3(2.9)
8. Hop.	0–10	1	3	5	3.4(2.9)
2. Landing after jumping	0–10	1	3	5.5	3.7(2.8)
5. Single leg jump.	0–10	1	3	6	3.8(3.1)
11. Jumping on a Trampoline	0–10	1	3	7	3.8(3.0)
6. Sliding	0–10	2	5	8	4.9(3.3)
4. Lateral Lunging	0–10	2.5	5	7.5	5.0(3.3)
7. Bring to a halt.	0–10	3	5	8	5.2(2.9)
12. Pivoting movement.	0–10	3	7	9	6.1(3.0)

Item scores: *0* not harmful, *10* extremely harmful

The different tasks showed a large variation in average scores ranging from 1.9 for Squatting (photo 3) to 6 for Pivoting (photo 12). Patient’s scores on the items Squatting and Lunging were extremely skewed with >50% reporting 0 or 1. These two items were eliminated from further analysis. All inter-item correlations were significant (ranging from .44 to .80). KMO measure of sampling adequacy was good (0.94), and Bartlett’s test of sphericity was significant (*p* < .0001). Entering the 10 PHOSA-ACLR items in the Principal Component analysis with Varimax rotation, resulted in the selection of 1 dimension with an Eigenvalue of 6.9, explaining 70% of the variation in the item scores (Hypothesis 1). The PHOSA-ACLR score is computed by averaging the remaining 10 items and is 4.2 (SD 2.6). Internal consistency of the PHOSA-ACLR is high (Cronbach’s Alpha = .95). The correlation between each item and the scale score is >.65. Score distribution on the scale shows univariate normal distribution.

*Criterion validity* and *hypothesis testing* is done in data from patient sample 1. Table 3 describes the sample characteristics of the participating patients.

**Table 3:** Sample characteristics of the Internal Consistency and reliability sample (*N* = 58)

	Range in observed scores	Respondents (%) with Maximum Functional score	Mean (SD)
Gender (% male/% female)	43%/57%		
Age (years)	18–53		25.9 (8.2)
Duration since ACLR in months	3–36		15.5 (8.0)
Lysholm (0–100)	12–100	2%	76.4 (2.2)
KOOS Pain (0–100)	33–100	19%	81.8 (17.7)
KOOS Other Symptoms (0–100)	29–100	2%	63.2 (12.8)
KOOS ADL (0–100)	35–100	36%	89.1 (14.2)
KOOS Sports and Leisure Activities (0–100)	00–100	7%	59.1 (30.6)
KOOS QoL (0–100)	6.3–75	0%	48.3 (14.1)
TSK (17–68)	20–51		35.75 (7.1)

*N* number of participants, *SD* Standard Deviation, *KOOS* Knee injury and Osteoarthritis Outcome Scale, *ADL* Activity of Daily Life, *QOL* Quality of Life, *TSK* Tampa Scale of Kinesiophobia

The average score of 76 on the Lysholm indicates a fair level of self-reported knee functioning. However, the substantial variety of scores on the Lysholm indicates that this sample includes both patients with poor and excellent scores. Average KOOS Pain and ADL scores indicate that on average patients perceived little pain and reported good function in ADL. In this sample, 36% of the patients reported the maximum score in functioning in ADL. Lower average scores were observed on the other KOOS subscales. Compared with the other KOOS scales, the average scores on Quality of life had the lowest value, with none of the participants scoring an optimal score (100). Finally, the average score on the TSK was high, with 38% of the participants showing FoH as indicated by a TSK score exceeding 37 points. In this sample PHOSA-TSK is correlated strongly with TSK score (*r* = .57, *p* < .001) (Table 1: Hypothesis 2). Patients reporting higher levels of kinesophobia assessed with the TSK also tended to report higher levels of FoM as assessed using the PHOSA-ACLR. Table 4 gives Pearson correlation calculated between all the variables in the study and both measures of FoH ((TSK and PHOSA-ACLR).

**Table 4:** Correlation of demographics and ACLR related variables with FoH, assessed with TSK and PHOSA-ACLR (*N* = 58)

	TSK	PHOSA-ACLR
	r 95%CI	r 95%CI
Age	−.23 (−.45,.04)	−.21 (−.39,.02)
Gender	−.01 (−.29,.24)	.20 (−.07,.49)
Months since reconstruction	−.09 (−.38,.11)	−.06 (−.34,.17)
Lysholm	−.57** (−.73,.46)	−.60** (−.79,-30)
KOOS symp	−.22 (.07,-.48)	−.30* (−.04,-.53)
KOOS pain	−.59** (−.36,-.74)	−.60** (−.38,-.77)
KOOS ADL	−.56** (−.35,-.71)	−.62** (−.35,-.79)
KOOS Sport	−.58** (−.36,-.75)	−.60** (−.37,-.78)
KOOS QoL	−.54** (−.32,-.71)	−.41** (−.18,-.62)
KSES ADL	−.64** (−.78,-48)	−.65** (−.81,-.37)
KSES Sports	−.54** (−.71,-.32)	−.68** (−.83,-.51)
KSES knee function	−.66** (−.79,-.54)	−.67** (−.82,-.39)
KSES future	−.66** (−.75,-.30)	−.53** (−.75,-.29)
MHLC intern	.30* (−.04,.51)	.10 (−.26,.36)
MHLC extern	−.40** (−.59,-.20)	−.21 (−.45,.04)
MHLC physician	−.21 (−.43,.11)	−.12 (−.34,.10)

*p < .05; ** *p* < .01; *TSK* Tampa Scale of Kinesophobia, *PHOSA-ACLR* Photographic Sports Related Activities-Anterior Cruciate Ligament Reconstruction, *KOOS* Knee injury and osteoarthritis outcome scale, *ADL* Activities of Daily Life, *QoL* Quality of Life, *K-SES* Knee Self Efficacy Scales, *MHLC* Multidimensional Health Locus of Control

Age, gender and time since reconstruction were not correlated with both indicator of Fear of Harm in this group. With exception of the KOOS symptom scales, all knee functioning indices were strongly correlated with both indicators of FoH. Higher score on the Lysholm and the KOOS subscales indicate better function, and these scores are inversely related to FoM assessed with both the TSK and the PHOSA-ACLR. The four subscales of the K-SES scale showed large negative correlations with both measures of FoH. Internal and external Locus of Control showed significant correlations with TSK, but not with PHOSA-ACLR. To determine if the observed correlation between PHOSA and TSK (Table 3) is significant after controlling for self-reported knee functioning, a stepwise regression analysis with TSK as dependent variable was performed. KOOS and Lysholm score together explained 42% of the variation in TSK (F = 6.3, df 6,52, *p* < 001). Entering the PHOSA-ACLR score in step 2 resulted in a 5% increase in variance explained (F = 5.1, df 1,50, *p* < .02).

*Reliability* and *Standard Error of Measurement* of the PHOSA-ACLR were assessed in 55 patients who completed the questionnaire twice with an interval of 1 week. Most participants were males (62%), and average age was 27.2 years (SD 9.5). Average time since reconstruction was 10 weeks (range = 4–35). Average item scores were 5.1 (SD 2.4) and 5.0 (SD 2.3) respectively. ICC between both assessments is .92 (95% CI .87,.96). The difference in average item score between both assessments was not significant (mean difference .10; SD 0.90). SEM is .90/√2 or .63.

*Hypothesis testing* showed that all but one hypothesis in Table 1 were confirmed.

## Discussion

The aim of this study was to describe the development of the PHOSA-ACLR and investigate its measurement properties. The photographs included in the PHOSA-ACLR depict sports related activities that may cause ACL ruptures, and therefore they are likely to induce FoH. Patients endorsed face validity of the items. The PHOSA-ACLR measures one dimension, FoH, and internal consistency of the scale is excellent. Criterion validity is supported by the strong positive correlation with TSK. As was expected, higher levels of impairment were related to higher levels of FoH, and low levels of knee related self-efficacy were associated with higher levels of FoH. The data in this study suggest excellent test-retest reliability of the PHOSA-ACLR. Overall, these findings indicate acceptable measurement properties of the PHOSA.

This study shows that the PHOSA-ACLR has some advantages compared to other measures to assess FoH in ACLR. The photographs are likely to appeal to the patient because they address their ACLR related fears. An important advantage of the PHOSA-ACLR is its unidimensionality: all items refer to FoH. In contrast, the TSK is less easy to interpret as it refers to at least 2 underlying dimensions: activity avoidance, and somatic focus [29]*.* Finally, the PHOSA-ACLR is specific for ACLR and can help the therapist to identify FoH for specific activities in an individual patient. Fear of Harm has been identified as one of the most important obstacles preventing athletes to return to previous levels of sports [15], and is related to diminished quality of life after ACLR [40]*.* Therefore, physicians and physical therapists should be able to recognise and address psychological factors that contribute to a patient’s postoperative decision to return to sport [41]*.* It is expected that the PHOSA-ACLR can help clinicians to identify patients with dysfunctional FoH after ACLR. These findings suggest that the PHOSA-ACLR has additional value as a diagnostic tool to determine perceived harmfulness of specific sports related activities in patients with ACLR.

However, there are some limitations regarding this study that need to be considered. Patients were not involved in designing the tool. However, patients in this study had few, if any comments on the content of the tool, and the items were considered valid for their situation. Furthermore, the study used convenience samples and therefore it is unclear if the results of this study are representative for all patients after ACLR. Data from several patient samples were obtained using an Internet tool guaranteeing anonymity. However, as a consequence we do not know how many patients were informed about the study, and how many patients refused to participate. This might constitute a selection bias. However, the 58 patient included in the reliability and validity sample have similar demographic characteristics compared to those reported in other studies on ACLR [30]. More importantly, a large range was found in the observed KOOS and Lysholm scores in the validity and reliability sample, indicating that patients with both poor and excellent self-reported knee function scores were represented. Another limitation of the study is that responsiveness to change of the PHOSA-ACLR was not determined in this study.Bearing these limitations in mind, it can be concluded that the PHOSA-ACLR seems to be an important additional tool to assess and evaluate FoH in ACLR. The instrument takes only some 5 min to complete, and face validity is endorsed by patients as they could relate to the depicted situation. The PHOSA-ACLR allows the assessment of an individual hierarchy of perceived harmfulness of different physical activities and movements specific for ACLR needed for graded exposure. Further research is needed to determine if the PHOSA-ACLR is responsive to change, and will be helpful to detect changes over time in FoH. Patients with high stable levels of FoH are likely to avoid high risk activities [42]*.* Exposure techniques based on the fear avoidance model, both in vivo and imagined, might help athletes to overcome FoH [18]*.* As yet, specific interventions directly targeting ACLR related FoH have not been reported [7]*.* Therefore, further studies are needed to show whether exposure techniques targeting FoH of harm in sports related activities in ACLR is effective to reduce fears and improve return to sports in these athletes and can only be answered in further clinical research.

## Conclusion

The PHOSA-ACLR is a valid and reliable instrument that allows the assessment of an individual hierarchy of perceived harmfulness of different physical activities and movements specific for ACLR. Such an individual assessment of perceived harmfulness is needed to be able to address fear of harm in therapeutic interventions based on graded exposure.

## Additioal file

Additional file 1: PHOSA-ACLR: Photograph Series of Sport Activities after Anterior Cruciate Ligament- Reconstruction. (DOCX 1399 kb)

## Abbreviations

ACL: Activities of daily life
ACL: Anterior cruciate ligament
ACLR: Anterior cruciate ligament reconstruction
FoH: Fear of harm
KOOS: Knee injury and osteoarthritis outcome scale
K-SES: KNEE self-efficacy scale
MHLC: Multidimensional health locus of control
PHODA: Photographic series of daily activities
PHOSA-ACLR: Photographic series of sport activities related to aclr
TSK: Tampa scale of kinesophobia

## References

[1] Majewski M, Susanne H, Klaus S (2006). Epidemiology of athletic knee injuries: a 10-year study. Knee.

[2] van Melick N, van Cingel RE, Brooijmans F, Neeter C, van Tienen T, Hullegie W, Nijhuis-van der Sanden MW. Evidence-based clinical practice update: practice guidelines for anterior cruciate ligament rehabilitation based on a systematic review and multidisciplinary consensus. Br J Sports Med. 2016;50(24):1506–15. 10.1136/bjsports-2015-095898 27539507

[3] Van Grinsven S, Van Cingel R, Holla C, Van Loon C (2010). Evidence-based rehabilitation following anterior cruciate ligament reconstruction. Knee Surg Sports Traumatol Arthrosc.

[4] Ardern CL, Taylor NF, Feller JA, Webster KE (2014). Fifty-five per cent return to competitive sport following anterior cruciate ligament reconstruction surgery: an updated systematic review and meta-analysis including aspects of physical functioning and contextual factors. Br J Sports Med.

[5] Ardern CL, Webster KE, Taylor NF, Feller JA (2011). Return to the preinjury level of competitive sport after anterior cruciate ligament reconstruction surgery: two-thirds of patients have not returned by 12 months after surgery. Am J Sports Med.

[6] Hewett TE, Di Stasi SL, Myer GD (2013). Current concepts for injury prevention in athletes after anterior cruciate ligament reconstruction. Am J Sports Med.

[7] Wierike S, Sluis A, Akker-Scheek I, Elferink-Gemser M, Visscher C (2013). Psychosocial factors influencing the recovery of athletes with anterior cruciate ligament injury: a systematic review. Scand J Med Sci Sports.

[8] Nyland J, Cottrell B, Harreld K, Caborn DN (2006). Self-reported outcomes after anterior cruciate ligament reconstruction: an internal health locus of control score comparison. Arthrosc: J Arthroscopic Related Surgery.

[9] Thomeé P, Währborg P, Börjesson M, Thomeé R, Eriksson BI, Karlsson J (2006). A new instrument for measuring self-efficacy in patients with an anterior cruciate ligament injury. Scand J Med Sci Sports.

[10] Chmielewski TL, Jones D, Day T, Tillman SM, Lentz TA, George SZ (2008). The association of pain and fear of movement/reinjury with function during anterior cruciate ligament reconstruction rehabilitation. J Orthop Sports Phys Ther.

[11] Ardern CL, Taylor NF, Feller JA, Webster KE (2012). Fear of re-injury in people who have returned to sport following anterior cruciate ligament reconstruction surgery. J Sci Med Sport.

[12] Everhart JS, Best TM, Flanigan DC (2015). Psychological predictors of anterior cruciate ligament reconstruction outcomes: a systematic review. Knee Surg Sports Traumatol Arthrosc.

[13] Flanigan DC, Everhart JS, Pedroza A, Smith T, Kaeding CC (2013). Fear of reinjury (kinesiophobia) and persistent knee symptoms are common factors for lack of return to sport after anterior cruciate ligament reconstruction. Arthrosc: J Arthroscopic Related Surgery.

[14] Czuppon S, Racette BA, Klein SE, Harris-Hayes M (2014). Variables associated with return to sport following anterior cruciate ligament reconstruction: a systematic review. Br J Sports Med.

[15] Kvist J, Ek A, Sporrstedt K, Good L (2005). Fear of re-injury: a hindrance for returning to sports after anterior cruciate ligament reconstruction. Knee Surg Sports Traumatol Arthrosc.

[16] Ardern CL, Taylor NF, Feller JA, Whitehead TS, Webster KE (2015). Sports participation 2 years after anterior Cruciate ligament reconstruction in athletes who had not returned to sport at 1 year a prospective follow-up of physical function and psychological factors in 122 athletes. Am J Sports Med.

[17] Wiese-Bjornstal DM, Smith AM, Shaffer SM, Morrey MA (1998). An integrated model of response to sport injury: psychological and sociological dynamics. J Appl Sport Psychol.

[18] Vlaeyen JW, Linton SJ (2012). Fear-avoidance model of chronic musculoskeletal pain: 12 years on. Pain.

[19] Miller, R.- Kori, S.- Todd, D. The tampa scale for kinisophobia. Unpublished report. 1991.

[20] Langford JL, Webster KE, Feller JA (2009). A prospective longitudinal study to assess psychological changes following anterior cruciate ligament reconstruction surgery. Br J Sports Med.

[21] Kugler K, Wijn J, Geilen M, de Jong J, Vlaeyen J. The Photograph series of Daily Activities (PHODA). CD-rom version 1.0. Institute for rehabilitation research and school for physiotherapy. Seattle: IASP Press; 1999.

[22] Leeuw M, Goossens ME, van Breukelen GJ, Boersma K, Vlaeyen JW (2007). Measuring perceived harmfulness of physical activities in patients with chronic low back pain: the photograph series of daily activities—short electronic version. J Pain.

[23] Mokkink LB, Terwee CB, Patrick DL, Alonso J, Stratford PW, Knol DL, Bouter LM, De Vet HC (2010). The COSMIN checklist for assessing the methodological quality of studies on measurement properties of health status measurement instruments: an international Delphi study. Qual Life Res.

[24] Ristić V, Ninković S, Harhaji V, Milankov M (2010). Causes of anterior cruciate ligament injuries. Med Pregl.

[25] Briggs KK, Lysholm J, Tegner Y, Rodkey WG, Kocher MS, Steadman JR (2009). The reliability, validity, and responsiveness of the Lysholm score and Tegner activity scale for anterior cruciate ligament injuries of the knee: 25 years later. Am J Sports Med.

[26] Roos EM, Roos HP, Lohmander LS, Ekdahl C, Beynnon BD (1998). Knee injury and osteoarthritis outcome score (KOOS)—development of a self-administered outcome measure. J Orthop Sports Phys Ther.

[27] De Groot IB, Favejee MM, Reijman M, Verhaar JA, Terwee CB (2008). The Dutch version of the knee injury and osteoarthritis outcome score: a validation study. Health Qual Life Outcomes.

[28] Goubert L, Vlaeyen JWS, Crombez G, Craig KD (2011). Learning about pain from others: an observational learning account. J Pain.

[29] Roelofs J, Goubert L, Peters ML, Vlaeyen JW, Crombez G (2004). The Tampa scale for Kinesiophobia: further examination of psychometric properties in patients with chronic low back pain and fibromyalgia. Eur J Pain.

[30] Thomeé P, Währborg P, Börjesson M, Thomeé R, Eriksson BI, Karlsson J (2008). Self-efficacy of knee function as a pre-operative predictor of outcome 1 year after anterior cruciate ligament reconstruction. Knee Surg Sports Traumatol Arthrosc.

[31] Beaton DE, Bombardier C, Guillemin F, Ferraz MB (2000). Guidelines for the process of cross-cultural adaptation of self-report measures. Spine.

[32] Wallston KA, Stein MJ, Smith CA (1994). Form C of the MHLC scales: a condition-specific measure of locus of control. J Pers Assess.

[33] Halfens R (1985). Locus of control. De beheersorientatie in relatie tot ziekte en gezondheidssgedrag. (locus of control in relation to illness and health behaviour).

[34] Terwee CB, Mokkink LB, Knol DL, Ostelo RW, Bouter LM, de Vet HC (2012). Rating the methodological quality in systematic reviews of studies on measurement properties: a scoring system for the COSMIN checklist. Qual Life Res.

[35] Cohen J (1992). A power primer. Psychol Bull.

[36] Williams B, Brown T, Onsman A (2012). Exploratory factor analysis: a five-step guide for novices. Australas J Paramedicine.

[37] Fleiss JL. Reliability of measurement. The design and analysis of clinical experiments. 1986;1–32.

[38] de Vet HC, Terwee CB, Knol DL, Bouter LM (2006). When to use agreement versus reliability measures. J Clin Epidemiol.

[39] Terwee CB, Bot SD, de Boer MR, van der Windt DA, Daniëlle AWM, Knol DL, Dekker J, Bouter LM, de Vet HC (2007). Quality criteria were proposed for measurement properties of health status questionnaires. J Clin Epidemiol.

[40] Filbay SR, Ackerman IN, Russell TG, Macri EM, Crossley KM (2014). Health-related quality of life after anterior cruciate ligament reconstruction: a systematic review. Am J Sports Med.

[41] Tjong VK, Murnaghan ML, Nyhof-Young JM, Ogilvie-Harris DJ (2014). A qualitative investigation of the decision to return to sport after anterior cruciate ligament reconstruction: to play or not to play. Am J Sports Med.

[42] Gignac MA, Cao X, Ramanathan S, White LM, Hurtig M, Kunz M, Marks PH (2015). Perceived personal importance of exercise and fears of re-injury: a longitudinal study of psychological factors related to activity after anterior cruciate ligament reconstruction. BMC Sports Sci Med Rehabil.

