# Supplementary material for: Measuring individual hierarchy of anxiety invoking sports related activities: development and validation of the Photographic Series of Sports Activities for Anterior Cruciate Ligament Reconstruction (PHOSA-ACLR)
Source: BMC Musculoskelet Disord. 2017 Jul 4;18:287. doi: 10.1186/s12891-017-1643-9 (PMC5496223; doi:10.1186/s12891-017-1643-9)
Supplement: Additional file 1: — PHOSA-ACLR: Photograph Series of Sport Activities after Anterior Cruciate Ligament- Reconstruction. (DOCX 1399 kb) [file 12891_2017_1643_MOESM1_ESM.docx]

**Instructions.**

We want to know which activities you consider harmful for the knee that was injured. Therefore, you will be shown a number of photographs depicting sports related movements.

- Imaging that you have to make the same movement with the injured knee as depicted in the photographs.
- Score each photograph at a scale from 0-10, with the score 0 depicting “not harmful at all”, and 10 “extremely harmful” for the knee.
- It is not important how painful you imagine the depicted movement to be. But how harmful you consider the movement to be.

1. **Running**

Score the activity depicted in the photograph below from 0 to 10, where 0 is “not harmful at all” and 10 is “extremely harmful”.


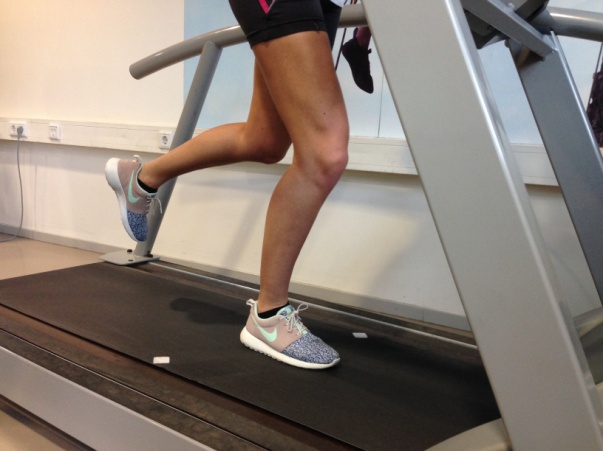


| 0 | 1 | 2 | 3 | 4 | 5 | 6 | 7 | 8 | 9 | 10 |
| --- | --- | --- | --- | --- | --- | --- | --- | --- | --- | --- |
|  |  |  |  |  |  |  |  |  |  |  |

**2. Landing after a jump**

Score the activity depicted in the photograph below from 0 to 10, where 0 is “not harmful at all” and 10 is “extremely harmful”.


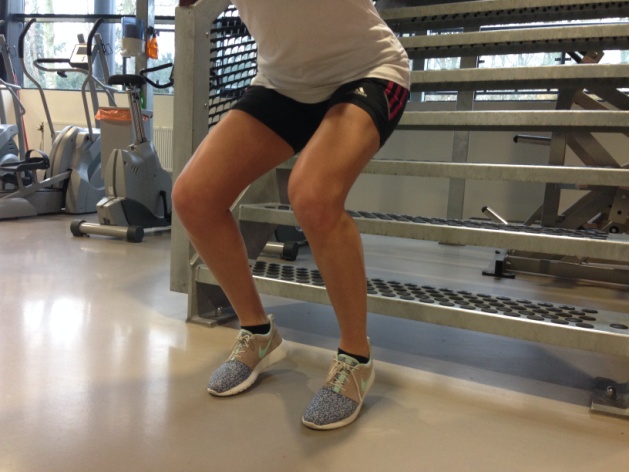


| 0 | 1 | 2 | 3 | 4 | 5 | 6 | 7 | 8 | 9 | 10 |
| --- | --- | --- | --- | --- | --- | --- | --- | --- | --- | --- |
|  |  |  |  |  |  |  |  |  |  |  |

3.**Squats**

Score the activity depicted in the photograph below from 0 to 10, where 0 is “not harmful at all” and 10 is “extremely harmful”.


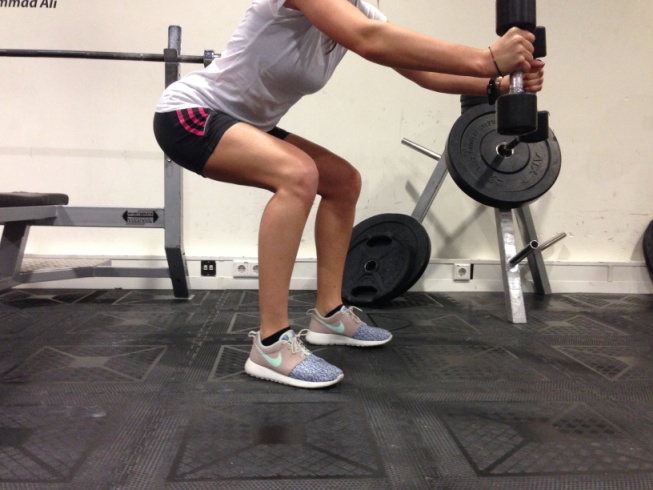


| 0 | 1 | 2 | 3 | 4 | 5 | 6 | 7 | 8 | 9 | 10 |
| --- | --- | --- | --- | --- | --- | --- | --- | --- | --- | --- |
|  |  |  |  |  |  |  |  |  |  |  |

4.**Lateral lunging**

Score the activity depicted in the photograph below from 0 to 10, where 0 is “not harmful at all” and 10 is “extremely harmful”.


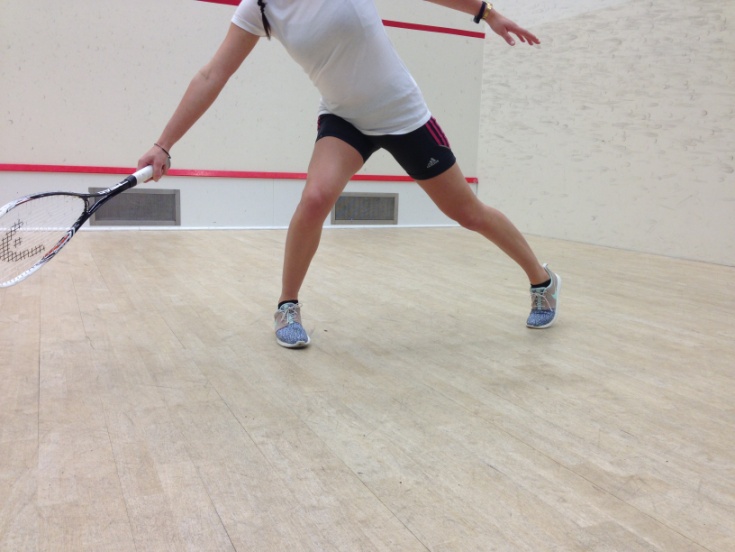


| 0 | 1 | 2 | 3 | 4 | 5 | 6 | 7 | 8 | 9 | 10 |
| --- | --- | --- | --- | --- | --- | --- | --- | --- | --- | --- |
|  |  |  |  |  |  |  |  |  |  |  |

**5.Single leg jump**

Score the activity depicted in the photograph below from 0 to 10, where 0 is “not harmful at all” and 10 is “extremely harmful”.

**
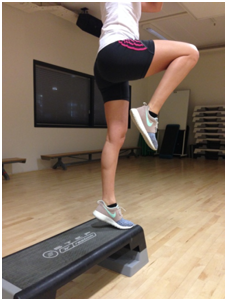
**

| 0 | 1 | 2 | 3 | 4 | 5 | 6 | 7 | 8 | 9 | 10 |
| --- | --- | --- | --- | --- | --- | --- | --- | --- | --- | --- |
|  |  |  |  |  |  |  |  |  |  |  |

**6.Sliding**

Score the activity depicted in the photograph below from 0 to 10, where 0 is “not harmful at all” and 10 is “extremely harmful”.


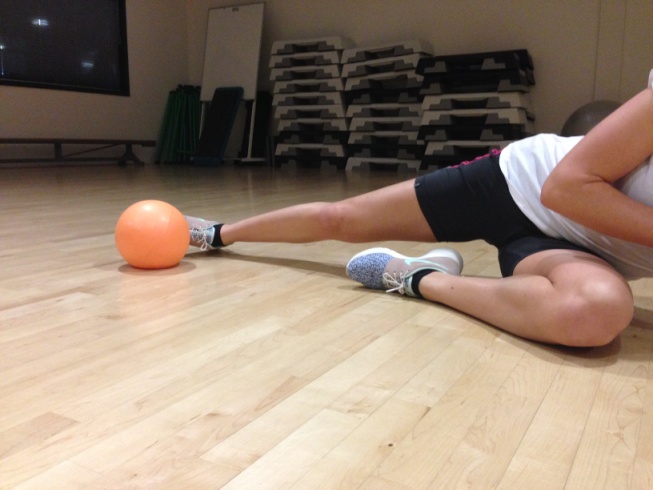


| 0 | 1 | 2 | 3 | 4 | 5 | 6 | 7 | 8 | 9 | 10 |
| --- | --- | --- | --- | --- | --- | --- | --- | --- | --- | --- |
|  |  |  |  |  |  |  |  |  |  |  |

**7.Sudden decelaration - stop**

Score the activity depicted in the photograph below from 0 to 10, where 0 is “not harmful at all” and 10 is “extremely harmful”.

 
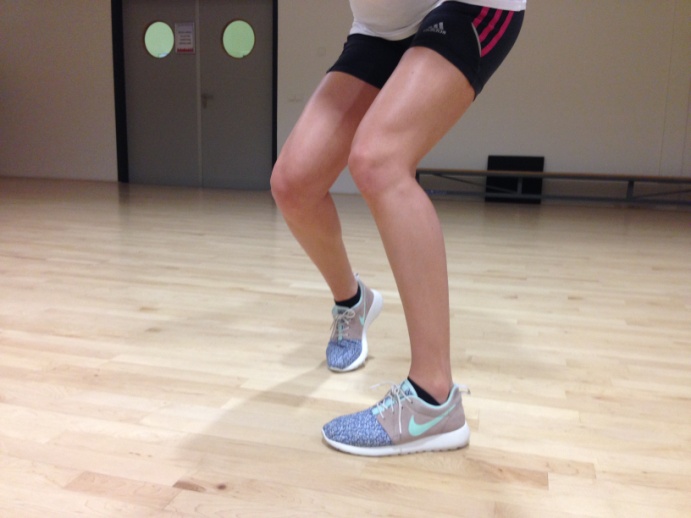


| 0 | 1 | 2 | 3 | 4 | 5 | 6 | 7 | 8 | 9 | 10 |
| --- | --- | --- | --- | --- | --- | --- | --- | --- | --- | --- |
|  |  |  |  |  |  |  |  |  |  |  |

**8.Hop**

Score the activity depicted in the photograph below from 0 to 10, where 0 is “not harmful at all” and 10 is “extremely harmful”.


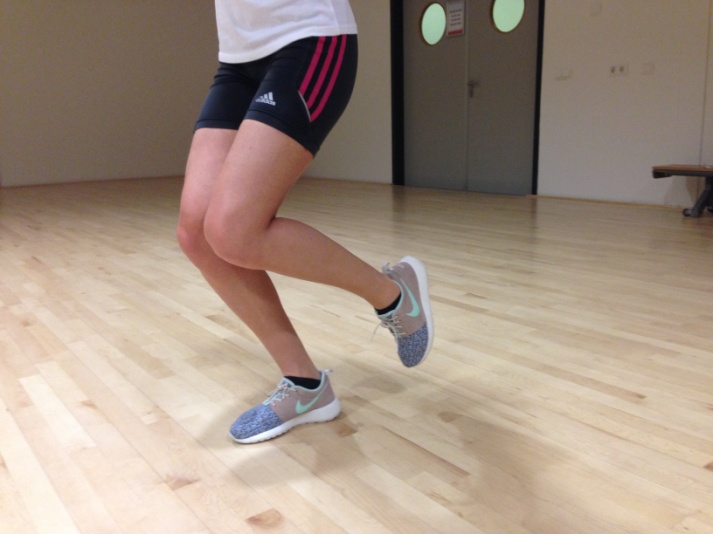


| 0 | 1 | 2 | 3 | 4 | 5 | 6 | 7 | 8 | 9 | 10 |
| --- | --- | --- | --- | --- | --- | --- | --- | --- | --- | --- |
|  |  |  |  |  |  |  |  |  |  |  |

**9.Lunge-front leg in injured leg.**

Score the activity depicted in the photograph below from 0 to 10, where 0 is “not harmful at all” and 10 is “extremely harmful”.

**
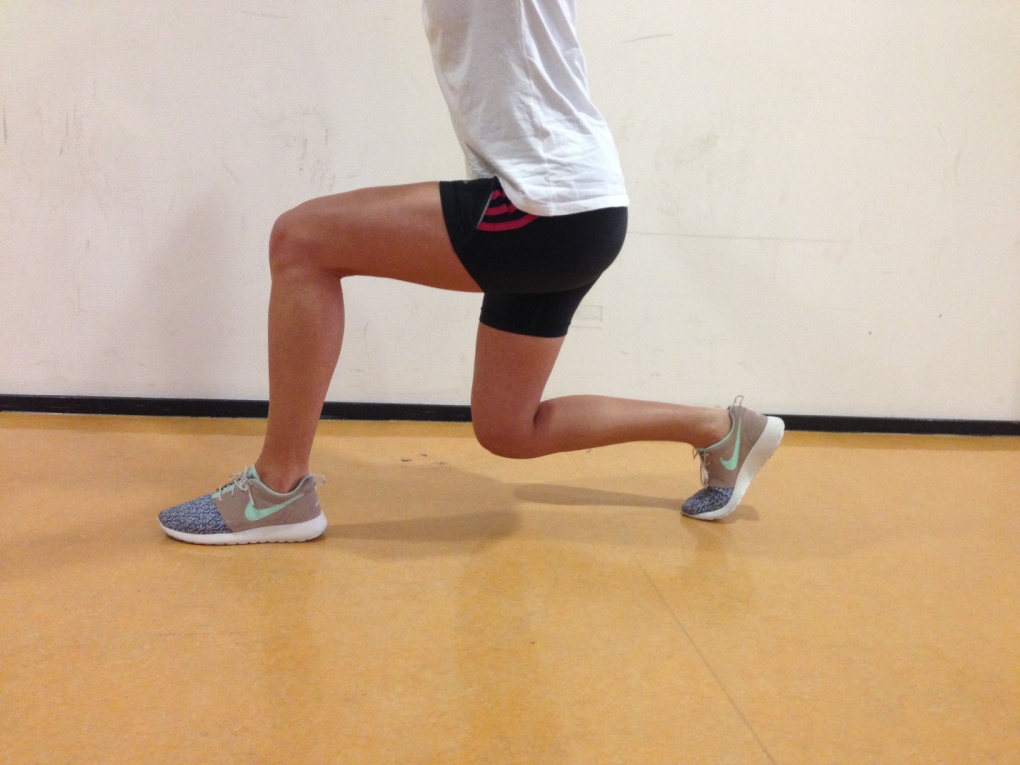
**

| 0 | 1 | 2 | 3 | 4 | 5 | 6 | 7 | 8 | 9 | 10 |
| --- | --- | --- | --- | --- | --- | --- | --- | --- | --- | --- |
|  |  |  |  |  |  |  |  |  |  |  |

**10.Start a sprint**

Score the activity depicted in the photograph below from 0 to 10, where 0 is “not harmful at all” and 10 is “extremely harmful”.


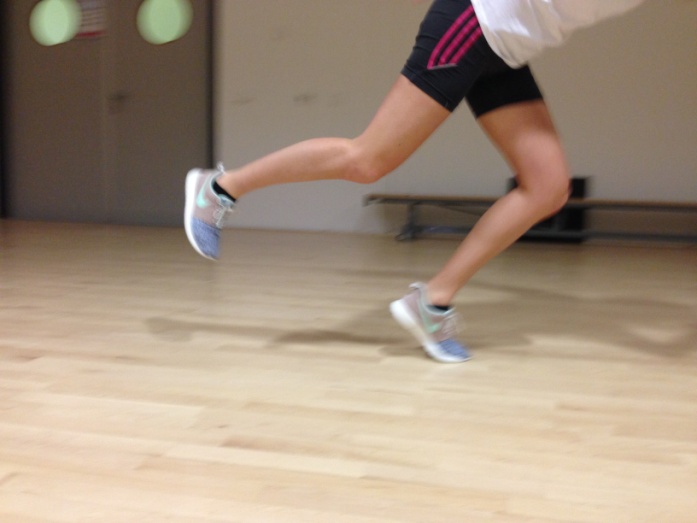


| 0 | 1 | 2 | 3 | 4 | 5 | 6 | 7 | 8 | 9 | 10 |
| --- | --- | --- | --- | --- | --- | --- | --- | --- | --- | --- |
|  |  |  |  |  |  |  |  |  |  |  |

**11.Jumping and landing on a trampoline.**

Score the activity depicted in the photograph below from 0 to 10, where 0 is “not harmful at all” and 10 is “extremely harmful”.

**
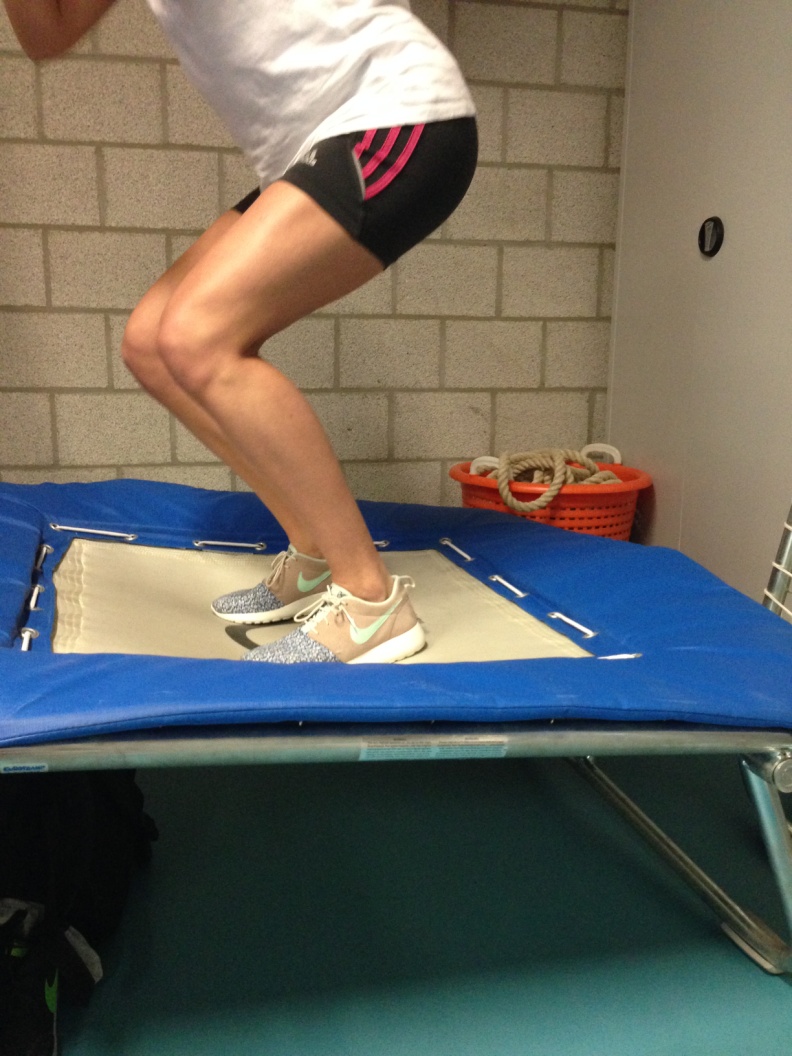
**

| 0 | 1 | 2 | 3 | 4 | 5 | 6 | 7 | 8 | 9 | 10 |
| --- | --- | --- | --- | --- | --- | --- | --- | --- | --- | --- |
|  |  |  |  |  |  |  |  |  |  |  |

**12.Pivoting movement**

Score the activity depicted in the photograph below from 0 to 10, where 0 is “not harmful at all” and 10 is “extremely harmful”.

| 0 | 1 | 2 | 3 | 4 | 5 | 6 | 7 | 8 | 9 | 10 |
| --- | --- | --- | --- | --- | --- | --- | --- | --- | --- | --- |
|  |  |  |  |  |  |  |  |  |  |  |

**
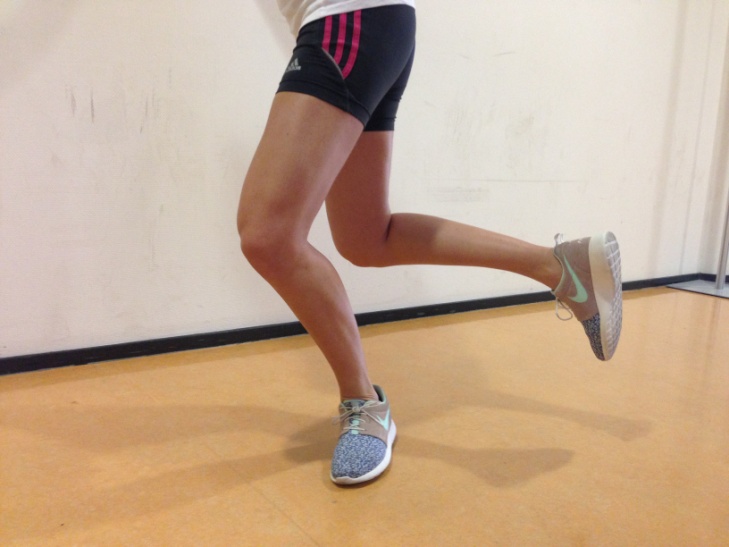
**
